# Complement Activation During Ischemia/Reperfusion Injury Induces Pericyte-to-Myofibroblast Transdifferentiation Regulating Peritubular Capillary Lumen Reduction Through pERK Signaling

**DOI:** 10.3389/fimmu.2018.01002

**Published:** 2018-05-23

**Authors:** Giuseppe Castellano, Rossana Franzin, Alessandra Stasi, Chiara Divella, Fabio Sallustio, Paola Pontrelli, Giuseppe Lucarelli, Michele Battaglia, Francesco Staffieri, Antonio Crovace, Giovanni Stallone, Marc Seelen, Mohamed R. Daha, Giuseppe Grandaliano, Loreto Gesualdo

**Affiliations:** ^1^Nephrology, Dialysis and Transplantation Unit, Department of Emergency and Organ Transplantation, University of Bari Aldo Moro, Bari, Italy; ^2^Department of Basic Medical Sciences, Neuroscience and Sense Organs, University of Bari Aldo Moro, Bari, Italy; ^3^Urology, Andrology and Renal Transplantation Unit, Department of Emergency and Organ Transplantation, University of Bari Aldo Moro, Bari, Italy; ^4^Veterinary Surgery Unit, Department of Emergency and Organ Transplantation, University of Bari Aldo Moro, Bari, Italy; ^5^Nephrology, Dialysis and Transplantation Unit, Department of Medical and Surgical Sciences, University of Foggia, Foggia, Italy; ^6^Division of Nephrology, Department of Internal Medicine, University of Groningen, University Medical Centre Groningen, Groningen, Netherlands; ^7^Department of Nephrology, Leiden University Medical Centre, Leiden, Netherlands

**Keywords:** complement system, pericytes, ischemia–reperfusion, tubulointerstitial fibrosis, capillary rarefaction, C1-inhibitor, C5a

## Abstract

Pericytes are one of the principal sources of scar-forming myofibroblasts in chronic kidneys disease. However, the modulation of pericyte-to-myofibroblast transdifferentiation (PMT) in the early phases of acute kidney injury is poorly understood. Here, we investigated the role of complement in inducing PMT after transplantation. Using a swine model of renal ischemia/reperfusion (I/R) injury, we found the occurrence of PMT after 24 h of I/R injury as demonstrated by reduction of PDGFRβ^+^/NG2^+^ cells with increase in myofibroblasts marker αSMA. In addition, PMT was associated with significant reduction in peritubular capillary luminal diameter. Treatment by C1-inhibitor (C1-INH) significantly preserved the phenotype of pericytes maintaining microvascular density and capillary lumen area at tubulointerstitial level. *In vitro*, C5a transdifferentiated human pericytes in myofibroblasts, with increased αSMA expression in stress fibers, collagen I production, and decreased antifibrotic protein Id2. The C5a-induced PMT was driven by extracellular signal-regulated kinases phosphorylation leading to increase in collagen I release that required both non-canonical and canonical TGFβ pathways. These results showed that pericytes are a pivotal target of complement activation leading to a profibrotic maladaptive cellular response. Our studies suggest that C1-INH may be a potential therapeutic strategy to counteract the development of PMT and capillary lumen reduction in I/R injury.

## Introduction

Ischemia/reperfusion (I/R) injury remains an unavoidable consequence after renal transplantation and the principal cause of delay graft function (DGF) (1). After brain death, the decreased blood flow induces a persistent rarefaction in peritubular capillaries (2, 3), whereas the following reperfusion exacerbates the pro-inflammatory response by activation of complement and coagulation (4). Pharmacologic treatments to prevent graft deterioration after I/R are currently lacking. Recently, genetic fate-mapping studies have identified pericytes as the major source of scar-forming myofibroblasts during progressive chronic kidney disease (5–8). Pericytes are mesenchymal-derived cells embedded in the capillary basement membrane and in direct contact with endothelial cells (9). Pericytes contribute to microvessel stability and show regeneration potential; renal pericytes regulate cortical and medullary flow by contracting or dilating in response to various stimuli released by the neighboring endothelial and tubular cells (10). Interestingly, data on cerebral ischemia showed that pericytes died “*in rigor*,” causing an irreversible constriction of capillaries that exacerbates the hypoxia (11, 12). Renal pericytes are PDGFRβ^+^/NG2^+^ cells that during their abnormal transdifferentiation in myofibroblasts upregulated the pro-fibrotic marker αSMA. The physiological role of the receptor tyrosin kinase PDGFRβ is to bind the platelet-derived growth factor B (PDGF-B) released by endothelial cells. The PDGFRβ signaling promoted the pericytes activation, migration, and the recruitment to the vascular wall of newly formed blood vessels. Nerve/glial antigen 2 (NG2) is a proteoglycan associated with pericytes during vascular morphogenesis (13).

Complement plays a pivotal role in renal I/R injury mediating tissue damage and amplifying innate and adaptive immune response (14–17). C1-esterase Inhibitor (C1-INH) blocks complement activation of the classical, lectin (14, 18, 19), and alternative pathways (20–22). Currently, C1-INH is used as treatment for hereditary angioedema (23), but several studies are evaluating the therapeutic potential in renal transplantation (24–26). In previous studies, we demonstrated that C1-INH is able to prevent the C5b-9 deposition along peritubular capillaries, limiting endothelial dysfunction and renal fibrosis during I/R (18, 27). The aim of our study was to investigate the involvement of complement in pericyte activation in the early phase of I/R injury.

## Materials and Methods

### Animal Models

Animal studies were carried out under protocol approved by Ethical Committee of the Italian Ministry of Health. Briefly, I/R was induced in pig by clamping the renal artery for 30 min followed by reperfusion, as described previously (18). A biopsy was performed before ischemia (T0). Pigs were divided into two groups: control (CTRL, *n* = 5, vehicle infused) and C1 Inhibitor treated group (C1-INH, *n* = 5). Five minutes before the beginning of the reperfusion, rhC1-INH was injected in the ear vein (500 U/kg). Biopsies were performed at 15, 30, and 60 min and 24 h after reperfusion. All animals were sacrificed 24 h after the procedure. Controlateral kidney was not removed for ethical concerns. A mouse model of renal bilateral I/R was performed in C5aR1^−/−^ mouse with C57BL/6 backgrounds, as previously described (28).

### Immunohistochemistry

Renal sections underwent deparaffination and heat-mediated antigen retrieval (citrate buffer, pH = 6.00) as previously described (18). For caspase3 and Ki67 detection, sections were permeabilized with Triton 0.25% for 5 min, then blocked by Protein Block Solution (DakoCytomation, USA) for 10 min. Incubation was performed with antibodies against: Caspase-3 (Novus Biologicals, Abingdon Science Park, UK), PDGFRβ (Abcam, Cambridge UK), Ki-67 (Novus Biologicals), and detected by the Peroxidase/DAB Dako Real EnVision Detection System (Dako, Glostrup, Denmark). The peroxidase reaction was shown by a brown precipitate, counterstained with Mayers hematoxylin (blue). Negative controls were prepared by incubation with a control irrelevant antibody. Images were scanned by Aperio ScanScope CS2 device and signals were analyzed with the ImageScope V12.1.0.5029 (Aperio Technologies, Vista, CA, USA).

### Analysis of Peritubular Capillaries Area

The peritubular capillaries area was calculated by Image J software. The cortical area of the entire biopsy acquired by Aperio ScanScope was analyzed in a stepwise fashion as a series of 10 consecutive fields, avoiding the arterioles, venules, and capillaries, which has a diameter upper than 50 µm. Values from all consecutive images for each biopsy were averaged.

### Cell Culture

Human placental-derived pericytes (PromoCell, Heidelberg, Germany) were grown in Serum Free Pericyte Growth Medium (PromoCell) at 5% CO_2_ and 37°C. Once they have reached the 70%, confluence cells were stimulated with human recombinant C5a (Biovision, San Francisco, CA, USA) at 10^−7^ M and human recombinant TGFβ-1 (10 ng/ml, Biovision). All experiments were performed at early P3–P5 passages. For pERK inhibition, cells were pretreated with SC1 (Pluripotin, Abcam) at 1–3–5 µM for 6–24 h, the cells were stimulated by C5a for indicated times. For C5aR inhibition assay, mouse monoclonal anti-C5aR (Abcam) was preincubated (1:10) for 1 h before the C5a exposition.

### Confocal Laser Scanning Microscopy

Renal sections and cultured pericytes were stained or double stained for αSMA (Santa Cruz Biotechnologies), PDGFRβ, NG2 (Abcam), C3 (HycultBiotech), and C5b-9 (Dako). For C3 and C5b-9 stainings, frozen kidney slides were used. For each experiment, 5 × 10^4^ cells were seeded on a cover slip, grown to 70% confluence, and fixed in 3.7% paraformaldehyde for 5 min. After blocking, slides were incubated with primary antibodies, overnight at 4°C and with secondary antibodies (Alexa Fluor, Molecular Probes, Eugene, OR, USA). TO-PRO-3 was used to counterstain nuclei. Negative controls were prepared by isotype control antibody. Image acquisition was performed with confocal microscope Leica TCS SP2 (Leica, Wetzlar, Germany).

### FACS Analysis

After incubations, cells were washed, detached by ice cold PBS-EDTA, permeabilized by Intraprep reagents (Instrumentation Lab), then incubated with FCR blocking reagent (Miltenyi Biotec) for 10 min at RT and with APC-conjugated anti-PDGFRβ (LSBio), FITC-conjugated anti-collagen I (Millipore, Millimarck) or mouse monoclonal anti-C5aR unconjugated (Abcam) for 20 min at RT in the dark. After washing, cells were re-suspended in FACS buffer. For apoptosis analysis, 5 × 10^5^ cells for each condition were washed with cold PBS 1× and double-stained with FITC-conjugated Annexin V/Propidium Iodide. Data were obtained using a FC500 flow cytometer (Beckmann Coulter) and analyzed by Kaluza software. Three independent experiments were performed. The area of positivity was determined using an isotype-matched mAb.

### MTT Assay

Cultured pericytes proliferation was measured by MTT Cell Proliferation Assay Kit, according the manufacturer instructions (Sigma-Aldrich). Briefly, 2 × 10^4^ cells/well were seeded in a 96-well plate, and then cells were treated with C5a, TGFβ1 (as indicated), PDGFBB (10 ng/ml) for 24 h.

### RNA Extraction and qPCR Analysis

RNA from pericytes was extracted using the miRNeasy Kit (Qiagen), 500 ng of total RNA was retrotranscribed with QuantiTect Kit (Qiagen). qPCR was carried out with SsoAdvanced™ Universal SYBR^®^ Green Supermix (Biorad) and the Light Cycler@96 (Roche). Primer list sequence in Table 1.

**Table 1:** List of primers used for qPCR.

Gene	Sequence 5′ → 3′
Connective tissue growth factor (CCN2)	F-TTGGCCCAGACCCAACTA R-GCAGGAGGCGTTGTCATT
C5aR1	F-GAGCCCAGGAGACCAGAACATG R-TACATGTTGAGCAGGATGAGGGA
ADAMTS1	F-GCACTGCAAGGCGTAGGAC R-AAGCATGGTTTCCACATAGCG
GAPDH	F-GAAGGTGAAGGTCGGAGTCA R-CATGGGTGGAATCATATTGGA

### Western Blot

Protein lysates were homogenized by RIPA buffer with phosphatase and protease inhibitors. Proteins (30 µg) were separated in 4–15% polyacrylamide gel and then transferred to PVDF membrane (0.2 mM) by Trans-Blot Turbo (BioRad, Hercules, CA, USA). After blocking in BSA at 5%, the membranes were incubated overnight with the following primary antibodies: pSMAD2/3 (Abcam), SMAD 2/3, pERK, extracellular signal-regulated kinases (ERK), Id2, matrix metallopeptidase (MMP9) (Santa Cruz Biotechnology, Inc.) and then with secondary antibody (hrp-conjugated, Santa Cruz). The same membrane was probed with mouse monoclonal anti-βactin antibody (1:20,000; Sigma). The electrochemiluminescence system was used to detect the antibody binding (Amersham, UK). The chemiluminescent signal was acquired by Chemidoc and quantified using Image J software.

### Statistical Analysis

Graphs were displayed using GraphPad Prism Software 5. Data were expressed as median ± interquartile range (IQR) and compared with a Mann–Whitney test for tissue immunostainings. For FACS, qPCR, MTT, and WB data were expressed as the mean ± SD. Statistical analysis was assessed using unpaired Student’s *t*-test. A *p-*value of <0.05 was considered significant.

## Results

### C1-INH Treatment Preserved Pericytes Phenotype After Renal I/R Injury

**Figure 1:**
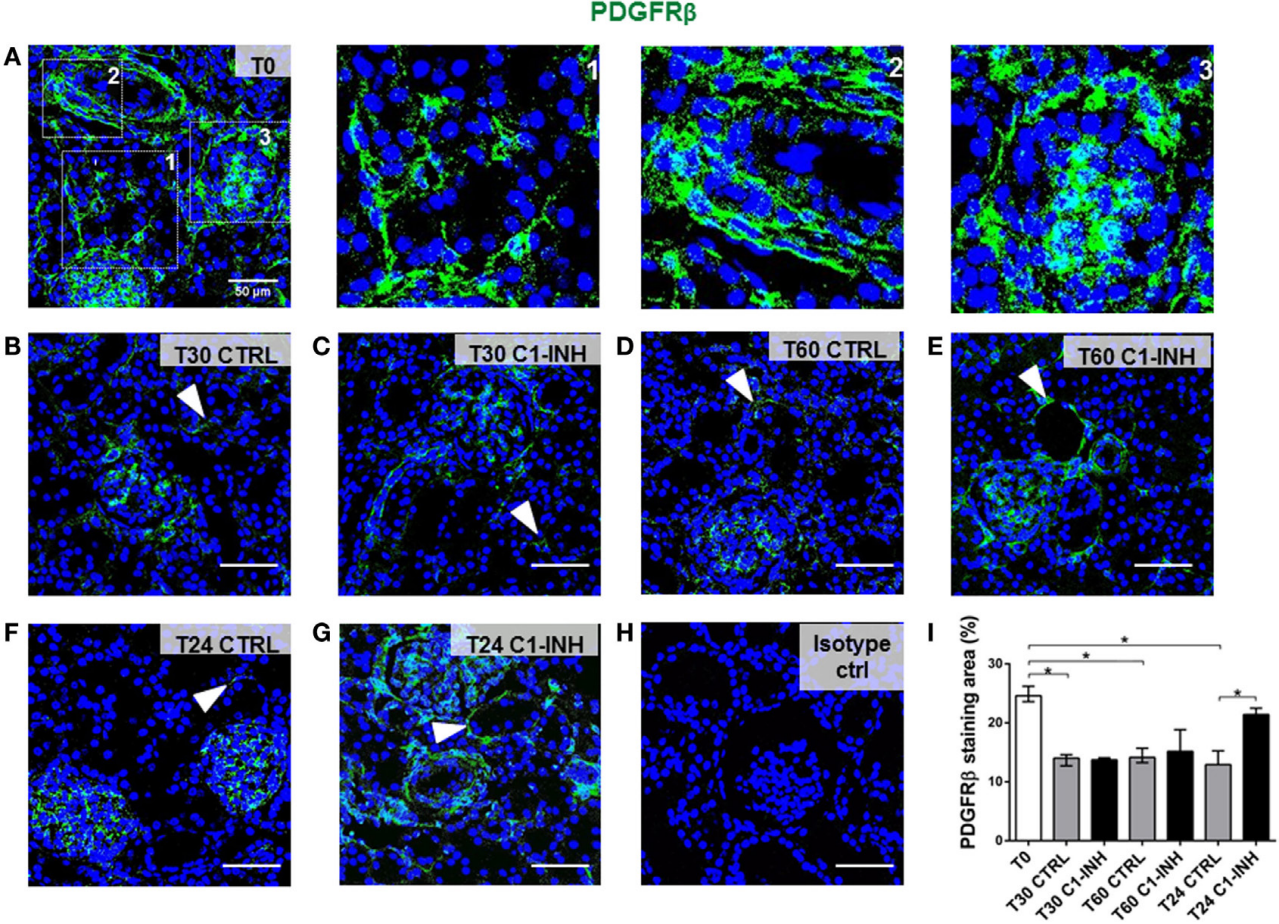
C1-INH treatment prevented PDGFRβ downregulation after I/R injury. PDGFRβ expression on paraffin sections at T0 (before ischemia) [**(A)**, zoomed in 1, 2, 3 at *right*], at 30 min **(B,C)**, 60 min **(D,E)**, and 24 h **(F,G)** after reperfusion. The groups analyzed were the CTRL **(B,D,F)** and C1-INH-treated pigs **(C,E,G)**. In the normal kidney **(A)**, PDGFRβ^+^ cells were localized in peritubular capillaries (1), in arterioles (2), in mesangial cells, and Bowman capsule (3). **(B–F)** Stainings showing that I/R injury induced the PDGFRβ downregulation in the peritubular areas, whereas 24 h of C1-INH treatment protected from pericytes loss. Arrowheads point to representative peritubular capillaries PDGFRβ^+^ cells. **(H)** Isotype control for PDGFRβ staining. Magnification 630×, scale bar = 50μm. **(I)** PDGFRβ^+^ staining area was expressed as the median ± interquartile range of five independent pigs for each group. **(F)** *p < 0.05.

To investigate the possible dysfunction of renal pericytes during I/R injury, biopsies were analyzed for the expression of PDGFRβ, a marker of pericyte (Figure 1). Under normal condition (T0, Figure 1A), PDGFRβ^+^ cells were detected in interstitial peritubular capillaries (Figure 1A, zoom1), in arterioles (Figure 1A, zoom2), mesangial cells, and Bowman’s capsule (Figure 1A, zoom3). I/R injury caused a significant decrease in PDGFRβ expression of pericytes in peritubular capillaries, a process that in the CTRL group began after 30 min and persisted until 24 h after reperfusion (Figures 1B,D,F). Treatment with C1-INH was unable to prevent early PDGFRβ downregulation (Figures 1C,E) but gave a significant preservation of peritubular PDGFRβ expression at 24 h after I/R injury (Figure 1G compared to Figure 1F). Notably, the PDGFRβ expression of mesangial cells was not significantly down regulated.

**Figure 2:**
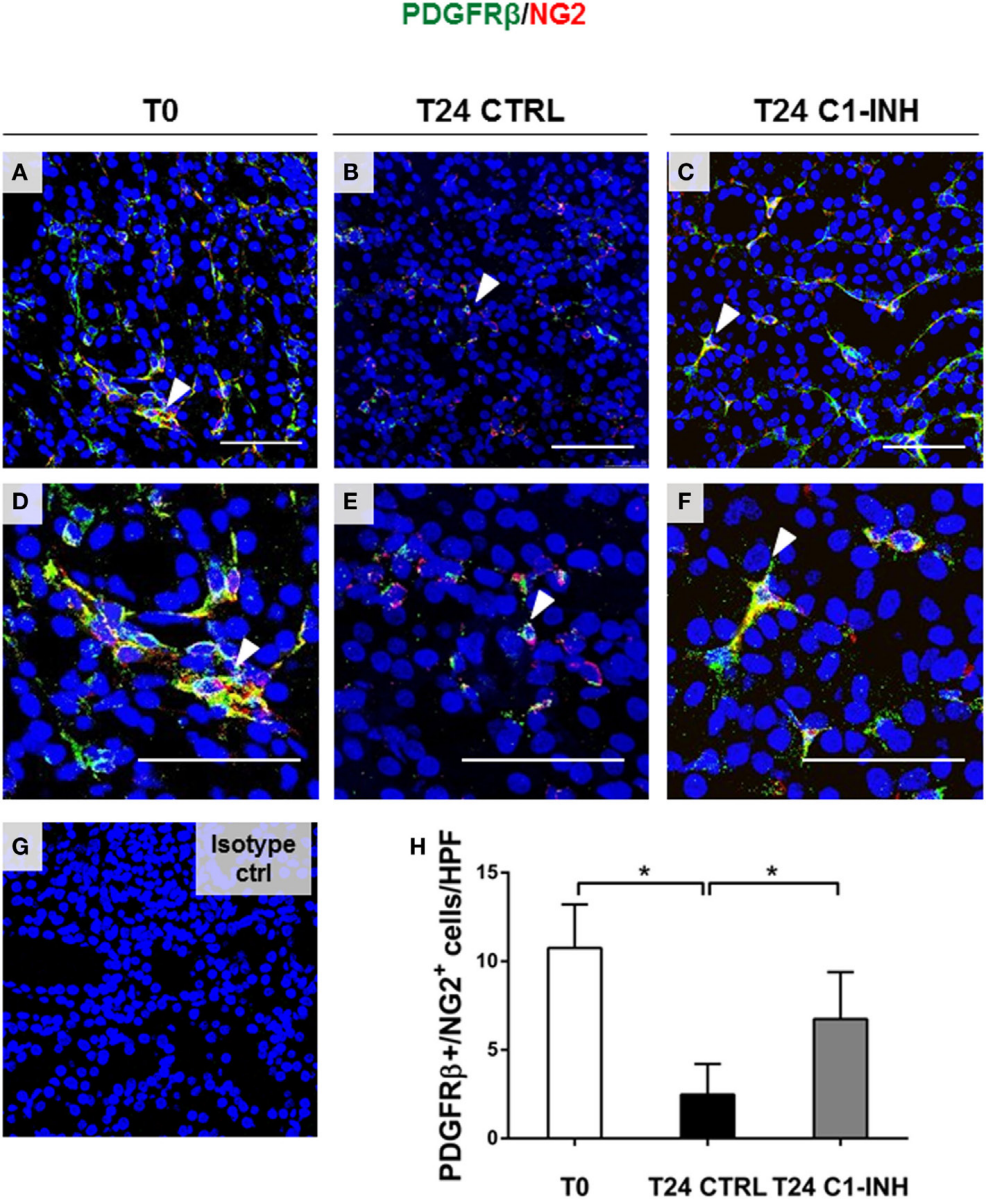
C1-INH preserved phenotype of double-positive PDGFRβ/NG2 pericytes after I/R injury. Renal tissues were double stained for the pericyte markers PDGFRβ (green) and NG2 (red). **(A,D)** Normal kidney, before ischemia (T0) showed the colocalization of the two signals in peritubular capillaries [arrowhead in **(A,D)** in merged images shown the overlap between PDGFRβ and NG2]. The number of peritubular capillaries-pericytes was reduced after 24 h of I/R **(B,E)**, in contrast, 24 h of C1-INH treated pigs showed PDGFRβ/NG2 marker restoration **(C,F)**. **(H)** Results are expressed as median ± interquartile range of the numbers of PDGFRβ^+^/NG2^+^ cells/high power fields (HPF) of five independent pigs for each group, *p < 0.05. Magnification, 630×, scale bar = 50 μm. **(G)** Isotype control staining for NG2.

Next, we used PDGFRβ and NG2 co-expression to specifically label pericytes (Figure 2). In swine kidney, pericytes markers were localized in the interstitial peritubular capillaries (Figure 2A). We found that all the perivascular NG2^+^ cells were PDGFRβ^+^; on the contrary, we found that mesangial cells were PDGFRβ^+^/NG2^−^. After 24 h of I/R, the total number of PDGFRβ/NG2 double positive pericytes significantly decreased; in accordance with Figure 1F, PDGFRβ^+^/NG2^+^ cells were barely detectable in the interstitial peritubular capillaries after I/R (Figures 2B,E). In contrast, C1-INH treated pigs were protected in pericytes phenotype as shown by a significant recovery in the number of PDGFRβ^+^/NG2^+^ cells in the interstitial peritubular capillaries regions. Interestingly, after 24 h from the C1-INH treatment, other PDGFRβ^+^/NG2^−^ cells (i.e., vascular smooth muscle cells) were protected from the PDGFRβ downregulation.

To further confirm the complement deposition at peritubular capillary level, we investigated the co-localization of PDGFRβ with C3 and C5b-9 after 30 min of reperfusion (Figures S1 and S4 in Supplementary Material) on frozen renal tissue. We found C3 and C5b-9 deposition around peritubular regions after 30 min; interestingly, C1-INH significantly counteracted this complement activation (Figures S1C–E and S4 in Supplementary Material).

### I/R Injury Did Not Affect Pericytes Viability *In Vivo*

**Figure 3:**
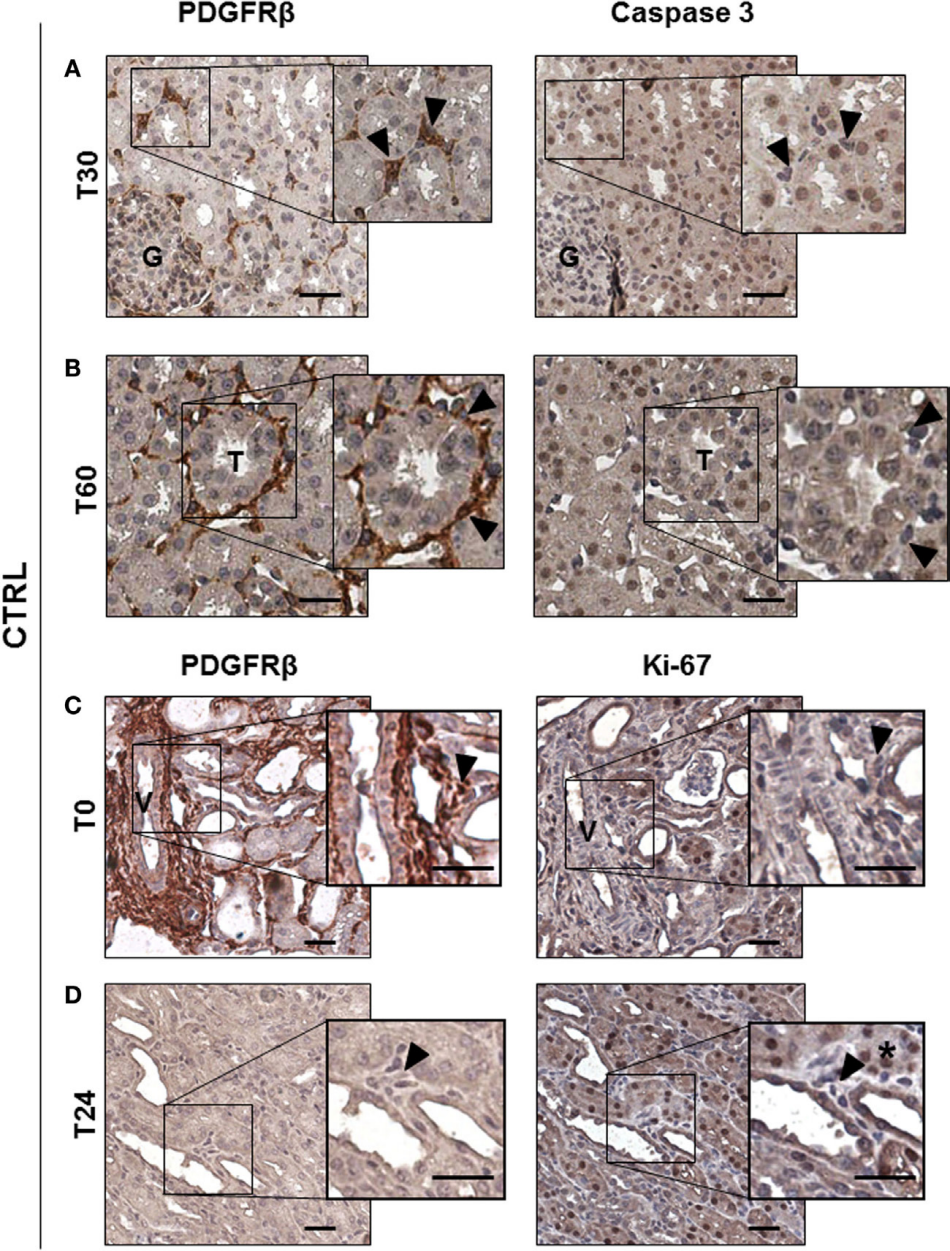
Ischemia/reperfusion injury did not induce pericytes apoptosis *in vivo*. IHC analysis of CTRL group serial sections labeled for PDGFRβ and Casp3 **(A,B)** and for PDGFRβ and Ki-67 **(C,D)**. PDGRβ^+^ peritubular capillaries-cells were detected by brown cytoplasmic staining. Apoptotic Casp3^+^-cells shown nuclear brown signal, while Casp3^−^ cells had blue nuclei. Double positive PDGFRβ^+^/Casp3^+^ peritubular pericytes were barely detected at early time after reperfusion [T30 in **(A)** and T60 in **(B)**], boxed area was enlarged at *right*. Magnification 20×, scale bar = 50 μm. Arrowheads indicate representative PDGFRβ^+^-pericytes (brown cytoplasmic cells) that showed negative Casp3 in the right panel. Immunohistochemical split diagram showing PDGFRβ^+^/Ki-67^−^ peritubular cells at T0 and T24h **(C,D)**. Arrowheads indicate representative PDGFRβ^+^-pericytes that did not show positivity for Ki-67 staining (blue nuclei) [**(C)**, *right*]; arrowheads indicate not proliferating perivascular cells [**(D)**, *right*]; asterisk indicates Ki-67^+^ tubular cells. G, glomeruli; T, tubuli; V, vessel.

To study the possible occurrence of pericyte apoptosis during I/R injury as observed in cerebral ischemia (12), we stained 30 min, 60 min, and 24 h serial sections for PDGFRβ and for the active form of Caspase 3 (Casp3) (Figure 3). After 30 and 60 min from reperfusion, no PDGRFβ/Casp3 double positive cells were detected in peritubular capillaries (Figures 3A,B arrowheads). As previously shown (18), apoptosis occurred predominantly in tubular epithelial cells (Figures 3A,B *right*, brown nuclei) (Figure S1A in Supplementary Material), and not in pericytes that were PDGFRβ^+^/Casp3^−^. In addition, we investigated whether renal pericytes proliferation could be detected, labeling serial sections for PDGFRβ and Ki-67, an antigen that marked nuclei in G1, S, and G2 cell cycle phases (29). Remarkably, 24 h after I/R injury, no Ki-67 positive cells could be found in interstitial peritubular capillaries (Figures 3C,D). In conclusion, in our model, cellular apoptosis and proliferation occurred at the level of tubular epithelial cells and not within cells of peritubular capillaries (Figure S1B in Supplementary Material).

### Complement Modulation Abrogated I/R Injury-Induced PMT and Attenuated Capillary Lumen Reduction

**Figure 4:**
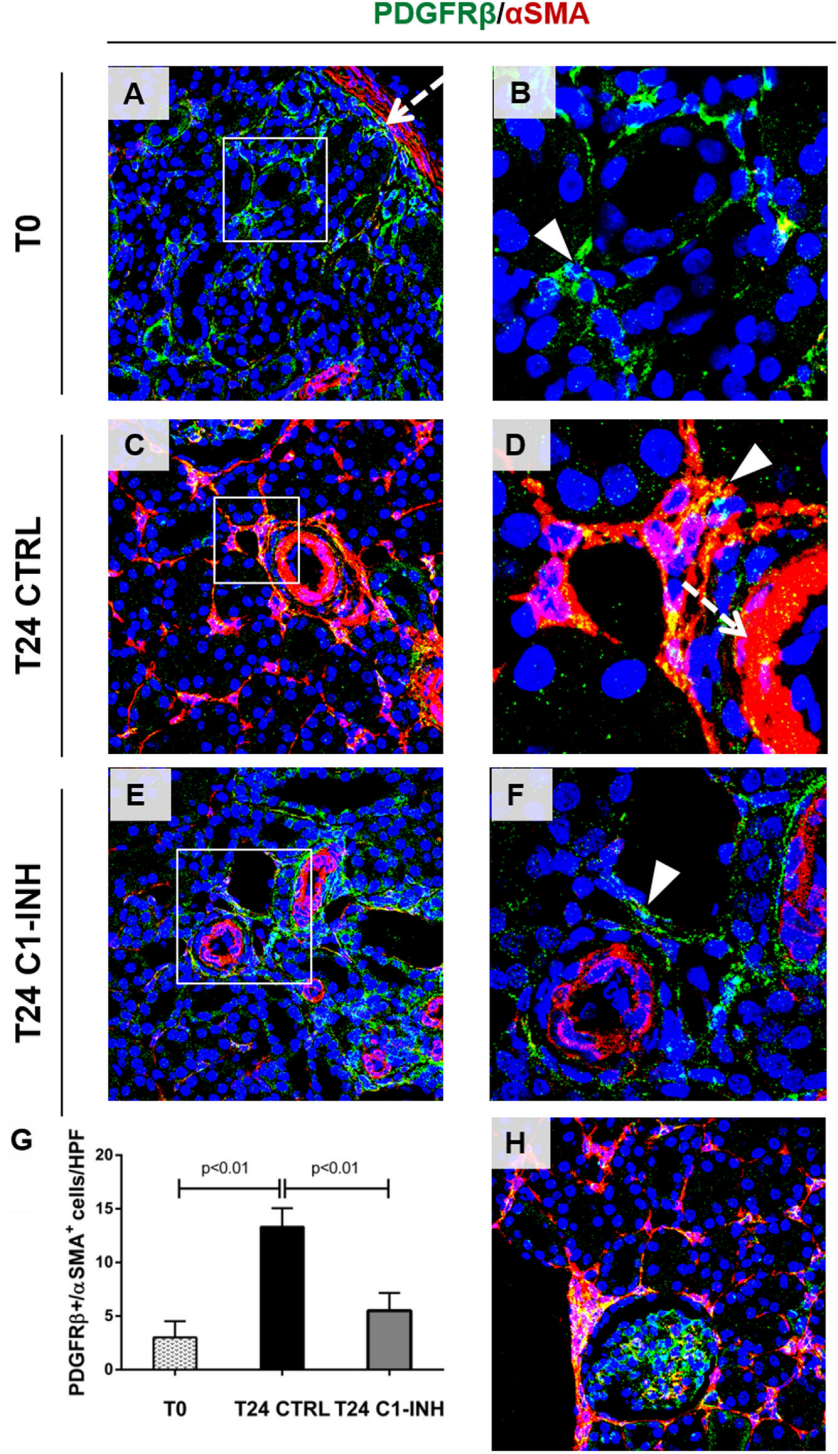
C1-INH significantly modulate the occurrence of PMT *in Vivo*. Confocal images showing interstitial peritubular capillaries pericytes co-labeled with PDGFRβ (green) and αSMA (red). In T0, PDGFRβ^+^/αSMA^+^ perivascular cells were barely detectable [**(A)**, boxed area was zoomed in **(B)**], and αSMA was localized in the wall of arteries [dotted arrows **(A,D)**]. After 24 h of ischemia/reperfusion injury [**(C)**, rectangle area was zoomed in **(D)**], the number of PDGFRβ^+^/αSMA^+^ cells increased predominately at peritubular capillaries level [**(D)**, arrowhead]. C1-INH treatment limited the αSMA increase **(E,F)**. The PDGFRβ decrease occurred at peritubular capillary level and did not affect the PDGFRβ^+^-mesangial cells [T24 CTRL in **(H)**]. Results are expressed as median ± interquartile range of the numbers of PDGFRβ^+^/αSMA^+^ cells/high power fields (HPF) of five independent pigs for each group **(G)**. Magnification, 630×.

**Figure 5:**
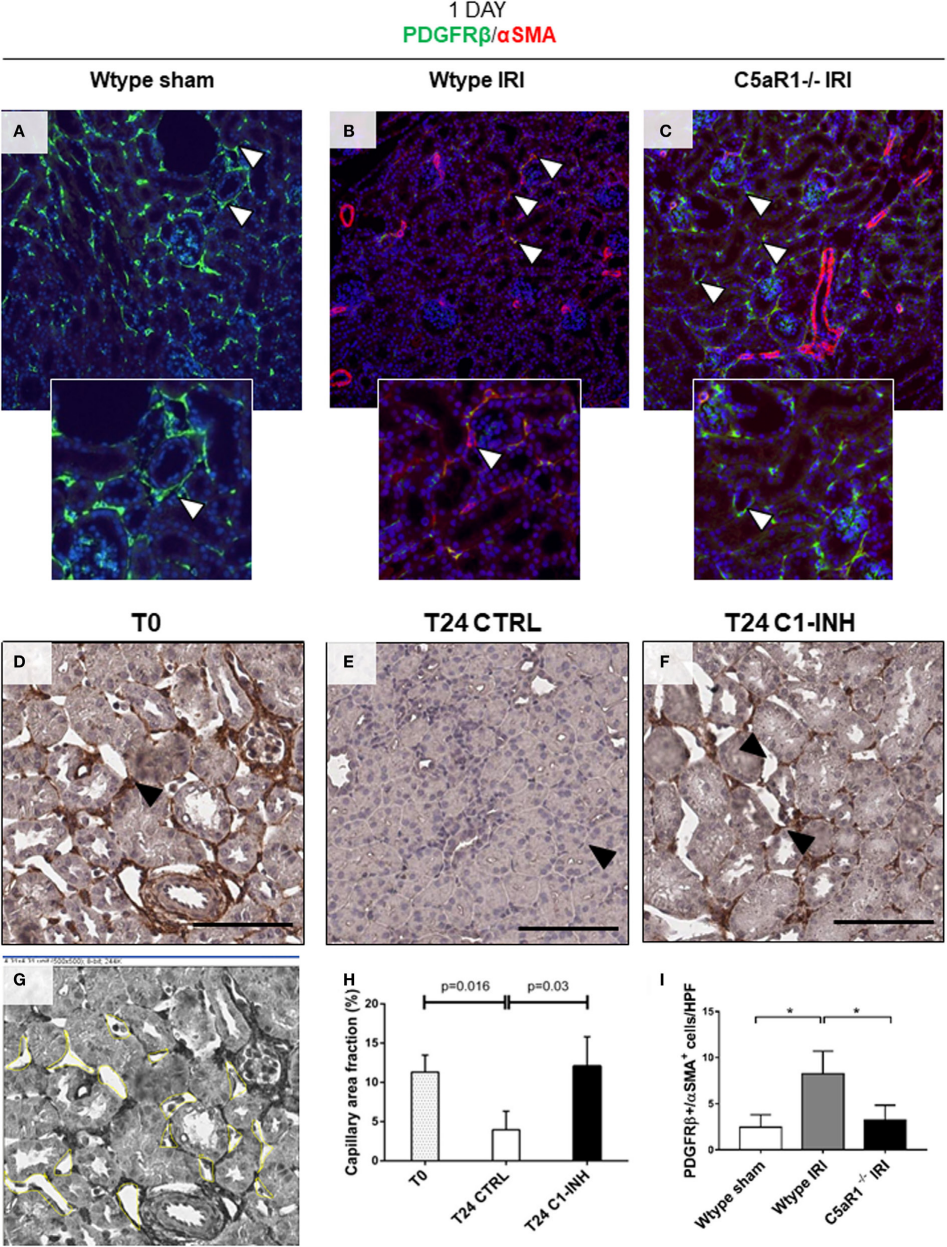
PMT is modulated by C5aR1 and associated with capillary lumen reduction 1 day after ischemia/reperfusion (I/R) injury. Renal I/R injury was performed for 24 h in C5aR1-deficient mice as showed in **(A–C,I)** and in a swine model treated with C1-INH as showed in **(D–H)**. **(A)** Confocal images showing interstitial peritubular capillaries pericytes in a mouse model of I/R injury co-labeled with PDGFRβ (green) and αSMA (red). In the Wtype sham, PDGFRβ^+^/αSMA^+^ perivascular cells were barely detectable. After 24 h of I/R injury **(B)**, the number of PDGFRβ^+^/αSMA^+^ cells increased predominately at peritubular capillaries level. **(C)** C5aR1^−/−^ mice were protected from PDGFRβ loss and αSMA increase. Results are expressed as median ± interquartile range (IQR) of the numbers of PDGFRβ^+^/αSMA^+^ cells/high power fields (HPF) of five independent mouse for each group **(I)**. Magnification, 50×. Representative IHC images of PDGFRβ-stained renal biopsies used to measure the peritubular capillaries area **(D,G)**. After 24 h of I/R injury **(E)**, microvessels appeared constricted with a significant decrease in luminal diameter. **(F)** The treatment with C1-INH restored basal capillary area fraction. Scale bar, 100 µM. **(G)** Schematic panel showing the calculation of capillary lumen area using Image J Software. **(H)** Graph indicating the mean of capillary lumen area. Results are expressed as median ± IQR of the capillary area fraction (%), *n* = 3 for each group.

Next, we investigated the possible occurrence of PMT after I/R. Normal kidney showed αSMA expression predominately in smooth muscle cells (wall of renal arteries, Figures 4A,D, dotted arrow). Before ischemia, we found PDGFRβ^+^/αSMA^−^ pericytes in interstitial peritubular capillaries (Figures 4A,B). After 24 h from I/R injury, perivascular cells upregulated αSMA together with an intense reduction in PDGFRβ expression, indicating PMT (Figures 4C,D). The co-localization of these two markers was more evident in arterioles and peritubular capillaries as shown in Figure 4D. Mesangial cells, which originate from the same FOXD1^+^ embryonic mesenchymal precursors of pericytes, expressed PDGFRβ (30, 31). However, PDGFRβ expression by mesangial cells remained unaffected and no increase of PDGFRβ^+^/αSMA^+^ was detected in glomerular cells (Figure 4H). C1-INH treatment significantly reduced the number of PDGFRβ^+^/αSMA^+^ cells in the peritubular capillaries, preserving the physiological pericytes phenotype (Figures 4E–G). These data demonstrate that that inhibition of C1 activity is associated with decreased PMT. Interestingly, we assessed the occurrence of PMT in a mouse model of renal I/R injury. In the Wtype sham, we detected the PDGFRβ expression at level of peritubular capillaries, where PDGFRβ^+^/αSMA^−^ cells were detected (Figure 5A). One day after reperfusion, a significant reduction of PDGFRβ and an increase of aSMA in the peritubular capillaries were observed in the Wtype (Figure 5B). On the contrary, C5aR1^−/−^ mice showed significantly lower number of PDGFRβ^+^/αSMA^+^ cells compared with the Wtype (Figures 5C,I).

To investigate the possibility that the occurrence of PMT might influence microvessel luminal diameter, capillaries lumen area was measured in PDGFRβ-stained renal sections (Figure 5G). Compared to T0 (Figure 5D), we found that PDGFRβ downregulation was evident in the interstitial capillaries characterized by lumen reduction (Figure 5E; % area fraction: T24CTRL 3.95 ± 2.36% versus T0 11.30 ± 2.6%). Interestingly, the treatment with C1-INH restored basal capillary area fraction (Figure 5F; T24 C1-INH 12.06 ± 3.8% versus T24 CTRL). We found that the restoration of capillary lumen was statistically significant (Figure 5H).

### C5a Induced PMT Without Affecting Pericytes Viability *In Vitro*

**Figure 6:**
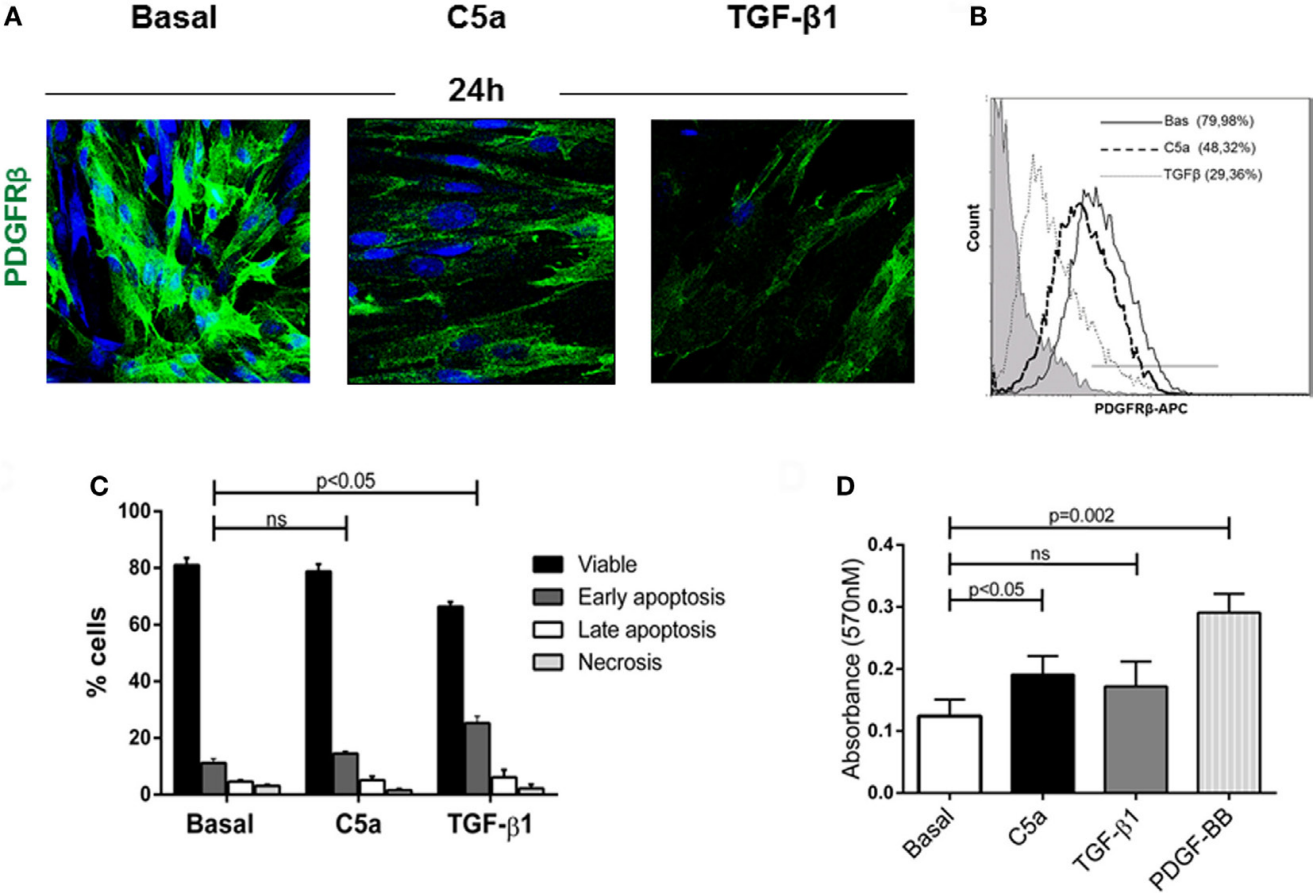
C5a induced PDGRFβ downregulation without affecting pericyte viability. **(A)** Immunofluorescence analysis showed the downregulation of PDGRβ after 24 h of treatment with C5a compared to basal. Magnification, 630×. **(B)** For FACS analysis; permeabilized cells were incubated with APC-conjugated PDGFRβ antibody. **(C)** After stimulation, pericytes were double stained with Annexin V-FITC and propidium iodide and analyzed to detect early, late apoptosis, and necrosis. The number of apoptotic cells was not increased. H_2_O_2_ 100 µM for 24 h was used as positive control (not showed). **(D)** Pericytes treated with C5a, TGFβ1, PDGF-BB (10 ng/ml), or medium alone were plated in 96-well plate and MTT assay was performed. Data represent the mean ± SD; *n* = 3.

To validate our findings *in vitro*, we next evaluated the PDGFRβ expression in pericytes culture stimulated with the complement anaphylotoxin C5a and TGFβ, a classic mediator of PMT (7, 32). After C5a stimulation, we found that the number of PDGFRβ^+^-pericytes significantly decreased indicating the phenotypic occurrence of PMT (Figures 6A,B PDGFRβ^+^ pericytes: C5a 48.32 ± 7.89 versus basal 79.98 ± 10.45%, *p* < 0.05). This activation persisted even after 48 h from stimulation (data not shown). By using the AnnexinV/Propidium Iodide and MTT test, we also found that pericytes did not undergo to early and late apoptosis upon C5a activation (Figure 6C, early apoptosis: C5a 14.57 ± 0.82% versus Bas 11.19 ± 1.52%, ns, Figure S2A in Supplementary Material) nor downregulated their proliferative response (Figure 6D). On the contrary, TGFβ stimulation upregulated the percentage of early apoptotic cells during pericytes activation.

**Figure 7:**
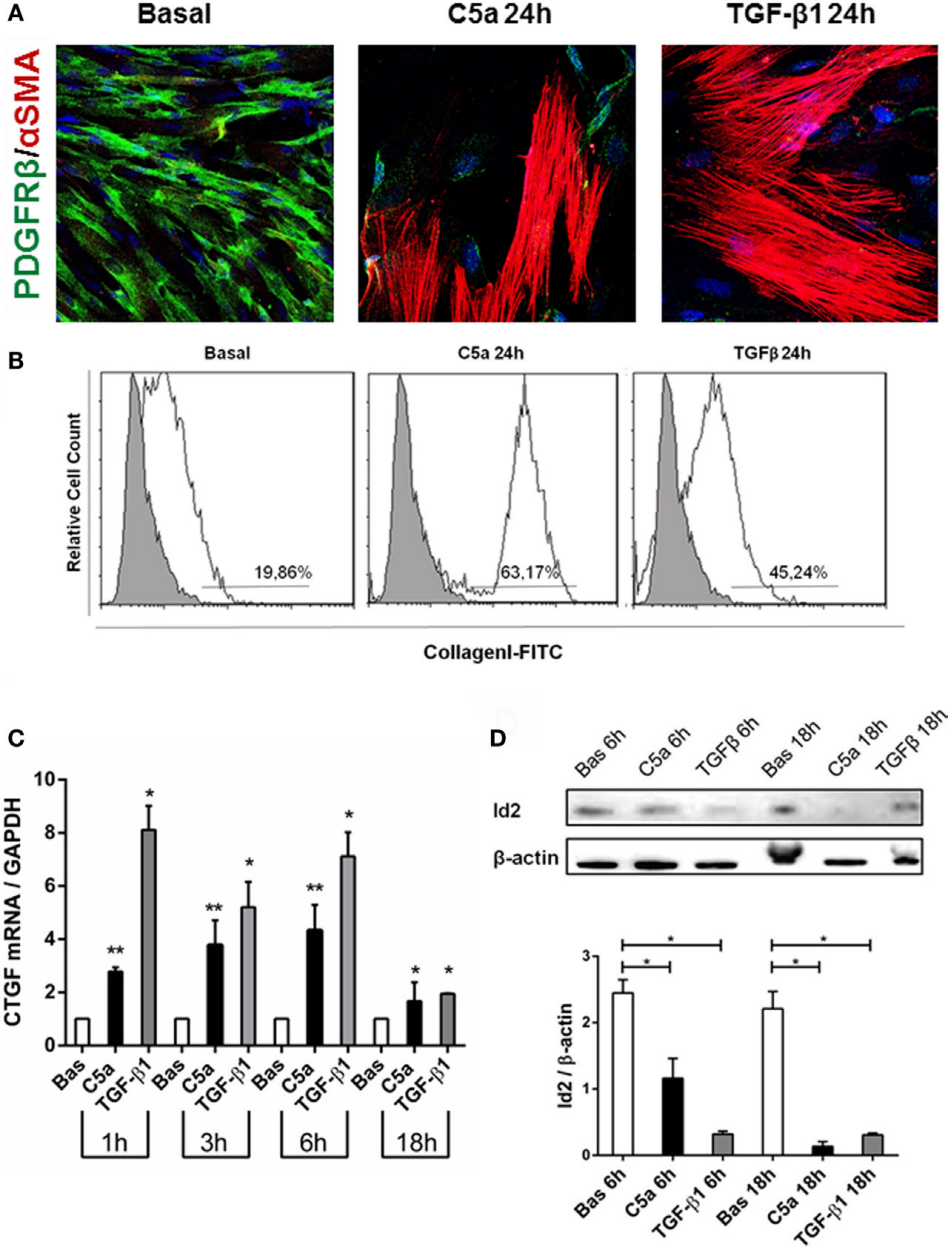
C5a induced PMT *in vitro*. Pericytes were incubated with C5a and TGFβ1 for 24 h. **(A)** Immunofluorescence showed a drastic remodeling of αSMA-stress fibers **(B)** FACS analysis showed increased collagen I expression in permeabilized cells, after C5a exposition. **(C)** mRNA expression levels of connective tissue growth factor (CTGF) (CCN2) were determined by qPCR. C5a stimulated pericytes showed a significant increase after 3 and 6 h of incubation. The fold change of CTGF expression was normalized to GAPDH. The histograms represent the mean ± SD, *n* = 3. **(D)** Western Blot showed a significant reduction of Id2 protein compared to basal condition, β-actin protein expression was used for normalization (*p < 0.05, **p < 0.01).

We also observed that C5a-stimulated pericytes acquired spindle-like shape morphology similar to fibroblasts. Performing immunofluorescence analysis, we found an increased expression of αSMA in stress fibers (PDGFRβ/αSMA, Figure 7A), indicating the acquirement of a contractile phenotype. Moreover, the morphologic changes were accompanied by increased collagen I protein synthesis (Figure 7B, C5a 24 h: 63.17 ± 8.22% versus basal: 19.86 ± 15.07%, *p* < 0.05) as well as connective tissue growth factor mRNA expression (Figure 7C). Interestingly, C5a increased metalloproteinase MMP9 protein level and ADAMTS1 gene expression, which are usually involved in pericytes detachment during PMT in experimental UUO (Figures S2B,C in Supplementary Material) (33).

Finally, we evaluated the modulation of Id2, a critical DNA-binding protein implicated in the regulation of the profibrotic/mesenchymal terminal differentiation (34–36). As expected, Id2 was highly expressed in normal cultured pericytes. By 6 h from C5a stimulation, we found a significant downregulation of Id2 in pericytes undergoing PMT; the downregulation was still detectable at 18 h and was comparable to the effects of TGFβ, a negative modulator of Id2 (Figure 7D).

### C5a Signaling Induces PMT by Canonical and Non-Canonical TGFβ Pathway *via* pERK

**Figure 8:**
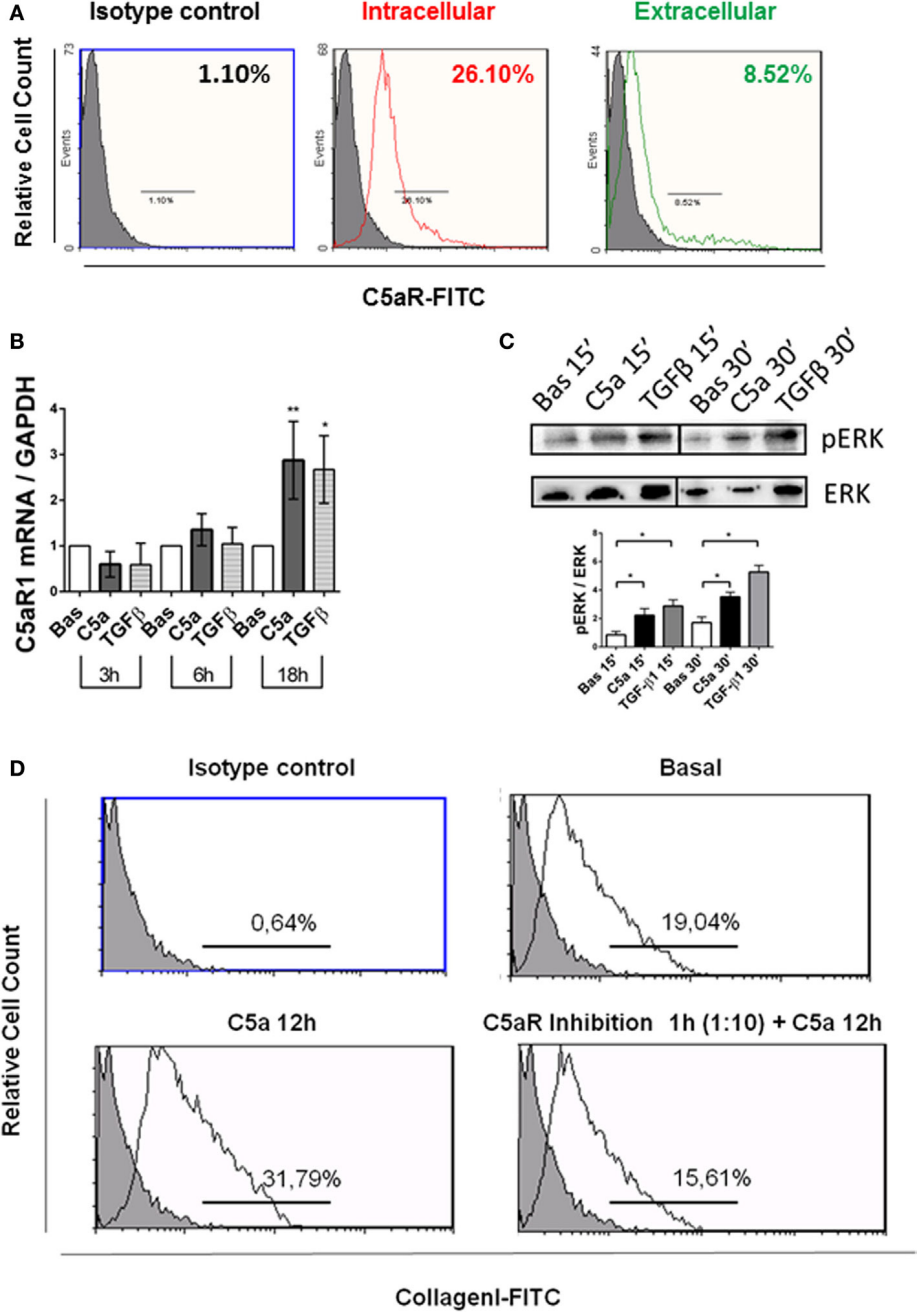
C5aR pathway activation contributes to PMT in human pericytes by extracellular signal-regulated kinases (ERK) phosphorylation. **(A)** Human pericytes expressed C5aR. Pericytes were assessed for presence of C5aR. FACS analysis showed both intracellular and extracellular C5aR expression in permeabilized (intracellular) and not permeabilized pericytes (extracellular) in basal condition. *n* = 3, MFI, mean fluorescence intensity. **(B)** qPCR analysis showed an increased expression of C5aR1 gene transcripts in pericytes after 18 h of C5a incubation. Expression levels were normalized by GAPDH. The histograms represent the mean ± SD, *n* = 3 (**p < 0.01, *p < 0.05, unpaired *t*-test versus basal for each time point). **(C)** Western blot of pericytes stimulated by C5a and TGFβ for 15 and 30 min. C5a increased the phosphorylation of ERK. Expression levels of pERK were normalized by total ERK (*n* = 3, *p < 0.05 versus basal). **(D)** FACS analysis of pericytes after C5aR blocking showing reduced collagen I production (*in bottom right*). Anti-C5aR (mouse monoclonal) was used to inhibit the binding of C5a to C5aR, before the C5a exposition. Basal (in *top, right*), C5a 12 h (in *bottom left*) *n* = 3, *p* < 0.05.

**Figure 9:**
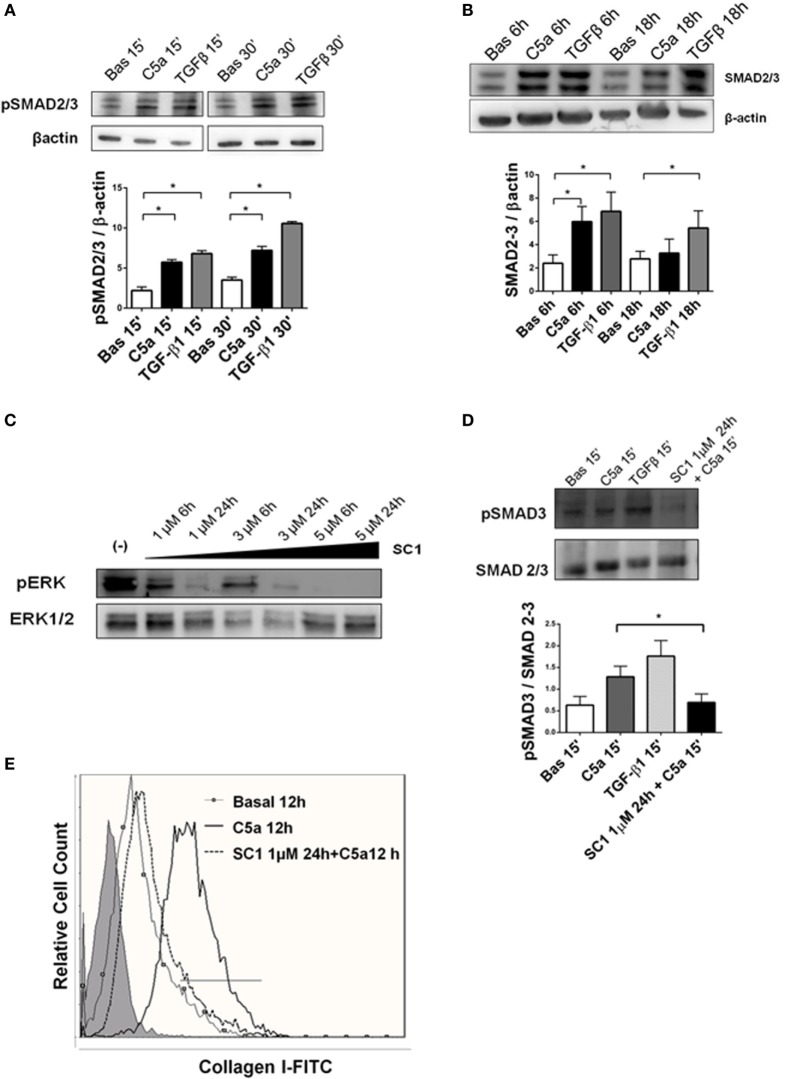
TGFβ canonical pathway is activated by C5a and modulated by pERK. **(A)** Western blot of pericytes stimulated by C5a and TGFβ. C5a increased the phosphorylation of SMAD 2/3 complexes after15 and 30 min **(B)** and upregulated the total SMAD 2/3 expression after 6 and 18 h. pSMAD2/3 and total SMAD 2/3-protein expression was normalized by β-actin. **(C)** Pericytes were cultured with or without SC1 (Pluripotin) pretreatment to inhibit extracellular signal-regulated kinases (ERK) phosphorylation. Time course of ERK phosphorylation at 6 and 24 h using increasing SC1 concentrations (1–3–6 µM) was performed. The relative density of the bands was normalized to total ERK. Pretreatment with SC1 1 µM for 24 h blocked C5a-induced SMAD3 phosphorylation after 15 min of incubation and the C5a-induced collagen I production after 12 h. **(D,E)**. Expression levels of pSMAD 3 were normalized by total SMAD. Graphs show mean ± SD, *n* = 3.

To identify the intracellular signaling involved in the C5a-induced PMT, cultured pericytes were incubated with complement anaphylotoxin and TGF-β1 (Figures 8 and 9). TGFβ contributes to renal fibrosis by the activation of canonical (SMAD 2/3) pathway and non-canonical [mitogen-activated protein kinase (MAPK)] pathways (37) (Figure S3 in Supplementary Material). Since also C5a, by interaction with the C5aR, induces the activation of ERK/MAPK pathway (38–40), we evaluated pERK protein modulation, as a possible common mediator of TGFβ and C5a signaling. We found by FACS analysis that pericytes expressed the C5aR at cytoplasmic and membrane level (Figure 8A). In addition, pericytes significantly upregulated the C5aR1 mRNA, which increased after 18 h of stimulation (Figure 8B). As shown in Figure 8C, C5a increased ERK phosphorylation after 15 and 30 min.

Next, we investigated whether the C5aR-induced signaling could play a role in promoting the PMT. We used anti-C5aR specific neutralizing antibody to inhibit the C5a binding to C5aR. Anti-C5aR prevented the increase of collagen I induced by C5a exposition (Figure 8D, *bottom right*). These results indicate that pericytes expressed the C5aR and that C5aR activation *via* pERK (non-canonical TGFβ pathway) might promote PMT. To further validate that pERK pathway was also pivotal in TGFβ-canonical signaling, we evaluated the effect of C5a stimulation on SMAD2/3 phosphorylation. First, we found the C5a increased the amount of pSMAD2/3 at 15 and 30 min (Figure 9A). Further time course at 6 and 18 h revealed that C5a led to a persistent increase of total SMAD2/3 complex (Figure 9B). Finally, we tested the effect of SC1 (Pluripotin) on C5a-induced PMT. SC1 is a dual kinase (ERK1, MAPK3) inhibitor that blocks ERK1/2 phosphorylation of at Thr-202/Tyr-204 (41). Analysis of ERK phosphorylation showed an inhibition at the concentration of 1 µM for 24 h (0.26 ± 0.18-fold compared to untreated cells 2.4 ± 1.18); higher concentrations (3–5 µM) interfered with cellular viability and were not considered. Pretreatment of pericytes with 1 µM SC1 for 24 h reduced ERK phosphorylation (Figure 8C), blocked C5a-induced SMAD3 phosphorylation at 15 min (SC1 1 µM SC1 for 24 h + C5a 15 min: 0.69 ± 0.32 compared to C5a 15 min: 1.32 ± 0.25) (Figure 8D) and significantly reduced the C5a-induced collagen I production at 12 h (SC1 1 µM SC1 for 24 h + C5a 12 h: 33.10 ± 12.15 compared to C5a 12 h: 8.34 ± 4.39) (Figure 8D, left bottom). These data support the role of C5a in promoting PMT by the activation of both TGFβ-canonical and non-canonical pathway.

## Discussion

In the present study, we demonstrated that inhibition of the complement system in I/R injury prevents the occurrence of PMT and the reduction of peritubular capillaries lumen areas. In particular, C5a had a pro-fibrotic activity driving pericytes toward a maladaptive dysfunctional phenotype by modulation of pERK activation.

Pericytes are mesenchymal-derived cells that interact with endothelial cells, releasing trophic factors such as VEGF and PDGF-BB (42). Recently, several studies demonstrated that pericytes are one of the myofibroblast precursors during development of tissue fibrosis (6, 43–45). Our results showed that complement is involved in transdifferentiation of pericytes in the early phases after kidney transplantation. Complement system is a key player in renal I/R injury (17, 46, 47), and C1-INH, a serine protease inhibitor used for the therapy of hereditary angioedema (23) might offer a new strategy for the prevention of I/R injury (25, 48, 49). Previously, it has been shown C1-INH treatment significantly prevents fibrosis, improves early and long-term renal function (26) and protected from TGFβ pathway activation (49). In our swine model of I/R, we found at early time after reperfusion (30 min) the deposition of complement components (i. e C4d, C3c, and C5b-9), along the peritubular capillaries (18), the areas where pericytes niche are localized. We also found the co-localization of PDGFRβ together with C3 (Figure S1 in Supplementary Material) and C5b-9 (Figure S4 in Supplementary Material). In accordance, starting from 30 min, the downregulation of PDGFRβ in our model began exactly along peritubular capillaries, without involvement of PDGFRβ expression in mesangial cells and larger arteries. The treatment with C1-INH, reduced the C4d peritubular deposition (18), and significantly restored pericytes markers expression, thereby accelerating the pericytes recovery. Even if pericytes identification requires transmission electron microscopy and fate-tracing analysis (5) several studies suggested that a PDGFRβ^+^ perivascular cell population is involved in collagen release, since specific PDGFRβ blocking reduced fibrosis development (32). Additionally, NG2 (50–52) was used to specifically label pericytes. We demonstrated a significant reduction in PDGFRβ/NG2 double positive cells after 24 h of I/R. Interestingly, our data are in line with Hosaka and colleagues (53) showing that in the PMT occurring during the tumor growth and metastasis, the loss of PDGFRβ and NG2 is not due to pericytes death nor proliferation. We found that renal I/R injury did not induce apoptosis of pericytes but their activation characterized by a maladaptive response not leading to cellular death but transdifferentiation toward a myofibroblast phenotype. In accordance, complement system did not affect pericytes viability *in vitro*. Furthermore, our data on renal pericytes are different compared with a model of cerebral ischemia *in vivo*, were pericytes died by apoptosis *in rigor mortis* and induced an irreversible constriction of micro vessel (12). Another difference in our data regarded the pericyte proliferation in the early stage of I/R. Using a rat model of I/R (54) and a transgenic reporter mice to determine the contribution of pericytes to fibrosis, previous reports described the increased proliferation of pericytes, starting from 48 to 72 h after injury (5). This difference might be explained by the fact that our analysis was conducted at 24 h; in addition, we analyzed a swine model.

Several studies revealed the importance of kidney pericytes for peritubular capillary integrity (55) and the microvascular rarefaction following ischemic acute kidney injury (AKI) (56, 57). After I/R, microvessels showed CD31 reduction and αSMA increase indicating the occurrence of endothelial-to-mesenchymal transition, which also contributes to kidney fibrosis (2, 27, 53); in this contest, αSMA, a marker of activated myofibroblasts, amplifies cell contractility with reorganization of stress fibers(9, 58). Here, we demonstrated that αSMA increase is also associated to microvascular pericytes. In accordance with Gomez and colleagues (59), we hypothesized that PMT might lead to direct constriction of vessels with reduction of capillaries density and lumen area. This process has been described in cerebral I/R injury where pericytes led to irreversible constriction of capillaries, exacerbating tissue hypoxia (11, 12). Interestingly, we found that treatment with C1-INH was capable to maintain the capillary lumen area by counteracting PMT. We recognized that C1-INH, beyond targeting classical and lectin pathway can inhibit the contact, coagulation, and fibrinolytic pathway involved in blood flow dysfunction (24). This can result in a reduced thrombi formation and a systemic improvement of vascular stability. However, PMT inhibition by C1-INH could provide a new mechanism to preserve graft from capillary rarefaction and reduction of lumen area.

Ischemia/reperfusion injury is primarily mediated by complement activation, with C5a playing a pivotal role also in inflammation response and allograft rejection (14, 15, 17, 60, 61). In this paper, we demonstrated for the first time that C5a can induce PMT with important pro-fibrotic effects. We stimulated cells with C5a because represents the most potent pro-inflammatory and chemotactic mediator (62) with specific receptors demonstrated at the level of renal resident cells. Nevertheless, despite the C5a pivotal pathogenic role, in clinical trials, the C5 inhibition by the human monoclonal antibody Eculizumab has been shown not to be sufficient to prevent DGF as well as antibody-mediated rejection (63, 64). Specifically, even after Eculizumab treatment, a residual C5 activity has been demonstrated (65). This could explain why not all patients benefit of an anti-C5 therapy in C3-mediated kidney diseases or during strong complement activation (24). Therefore, strategies that act upstream of C5 activation to prevent opsonization and generation of C3 activated products (i.e., C3a, iC3b, C3b, and later C5a) have been evaluated (24). As endogenous serine protease inhibitor, C1-INH has an excellent safety compared to Eculizumab (66, 67) and indirectly inhibits the release of the reactive late component C5a (68).

The C5a/C5aR pathway has extensively shown to cause recruitment of neutrophils and macrophages and exacerbate tubular injury in acute kidney injury. C5aR deficiency on renal cells or circulating leukocytes can significantly ameliorate renal injury (14, 16, 28, 39, 69).

The C5a-induced PMT was characterized by: the acquirement of αSMA stress fibers, the production of extracellular matrix proteins as collagen I, the downregulation of the antifibrotic BMP7-Id2 signaling (70–72), and finally by the activation of TGFβ canonical and non-canonical pathway (70–77). Our data connecting complement activation with fibrosis are in accordance with other disease model such as lung fibrosis where complement might lead to SMAD2/3 dependent and independent pathway activation, shifting the initial acute inflammatory response in a chronic profibrotic state (77).

We also analyzed ERK activation at early times after C5a stimulation, since ERK is a common downstream mediator of C5aR, TGFβ non-canonical signaling, and a possible inducer of TGFβ canonical pathway (73, 75) (Figure S3 in Supplementary Material). First, we showed that human pericytes expressed the C5aR, both at cytoplasmic level and on membrane surface. Second, by blocking the C5aR, we demonstrated that C5aR signaling is involved in collagen I release. As observed by other group (77), C5a can activate SMADs proteins, independently from TGFβ. In this signaling, ERK could act as bifurcation point to induce both the non-canonical and the canonical TGFβ pathways (78). After ERK phosphorylation blockade by SC1 (pluripotin), a dual kinase (ERK1, MAPK3) inhibitor (41), we found a significant reduction of C5a-induced SMAD3 activation and of C5a-induced collagen production. In accordance with recent evidences (79, 80), these results suggest that ERK might regulate TGF-β/Smad signaling. Therefore, next to TGFβ (81), also innate immune signaling (59) (i.e., anaphylotoxins) might lead to an amplification of interstitial extracellular matrix accumulation by generating myofibroblast *via* PMT after AKI (82). In accordance with our *in vitro* findings, the *in vivo* C5aR1 deficiency protected from PMT, indicating that C5a/C5aR1 is involved in tubulointerstitial fibrosis as shown by Martin et al. (83).

In conclusion, our data suggest that in the early phase of I/R injury, renal pericytes are a major target of complement activation resulting in maladaptive response and PMT. Considering the pivotal role of renal pericytes in preserving vascular homeostasis and maintaining blood perfusion, our data offer new insight into the pathogenic mechanisms regulating vascular capillary reduction and fibrosis development in AKI with potential future therapeutic application.

## Ethics Statement

The study was approved by the ethical committee of the Ministry of Health, Italy. This work was supported by University of Bari “Aldo Moro” Ministero della Salute (Ricerca Finalizzata 2009 granted to GC and GG) and an unrestricted research grant from Pharming Group.

## Author Contributions

GC, RF, AS, CD, FS, PP, MB, and FS performed the experiments design; RF, AS, CD, FS, and GC performed experiments; MB, AC, GL, and GS provided the pig animal model samples; MS and MD contributed the mouse model. GC supervised all the experiments. GG and LG supervised the project. All authors were involved in data interpretation. GC and RF wrote the paper, all authors had final approval of the submitted and published versions.

## Conflict of Interest Statement

The authors declare that the research was conducted in the absence of any commercial or financial relationships that could be construed as a potential conflict of interest.

## Supplementary Material

Figure S1 Ischemia/reperfusion (I/R) injury did not induce apoptosis or proliferation within perivascular compartment. Quantification of Casp3^+^ **(A)** and Ki-67^+^ **(B)** cells by IHC after I/R injury with distinction between tubular and perivascular cells. At different times from reperfusion, the Casp3^+^ and Ki-67^+^ were detected predominately at tubular level. Results are expressed as mean ± SD of Casp3^+^ or Ki-67^+^ cells/high power fields (HPF). Immunofluorescence images showing interstitial peritubular capillaries pericytes co-labeled with PDGFRβ (green) and C3 (red). In T0, PDGFRβ^+^/C3^+^ perivascular cells were barely detectable **(C)**. After 30 min of I/R injury [**(D)**, rectangle area was zoomed in **(G)**], the number of PDGFRβ^+^/C3^+^ cells increased predominately at peritubular capillaries level [**(D)**, arrowheads]. C1-INH treatment limited the C3 deposition **(E)**. Isotype control staining was used as negative control **(F)**. Results are expressed as median ± interquartile range of the numbers of PDGFRβ^+^/C3^+^ cells/HPF of five independent pigs for each group **(H)**. Magnification, 50×.

Figure S2 C5a did not trigger apoptosis and activated expression of matrix metallopeptidase (MMP9) and ADAMTS1 in pericytes. Human pericytes were treated with C5a and TGFβ for 6, 18, and 24 h. **(A)** Cell apoptosis or necrosis was analyzed by flow cytometry after Annexin V/propidium iodide (PI) staining. The units of the *Y* and *X* axes are fluorescence intensity. Early apoptotic cells are Annexin-V+/PI−; late apoptotic cells are both Annexin-V + 7/PI+; and necrotic cells were Annexin-V−/PI+. Data are expressed as apoptosis (early and late %) or necrosis (%) (as indicated in Figure 5C). **(B)** Western blotting demonstrated the increased expression of active form (85 kDa) of MMP9. **(C)** qPCR demonstrated the upregulation of ADAMTS1 mRNA after C5a and TGFβ exposition. *p* < 0.05 versus basal.

Figure S3 Schematic pathway showing the possible cross-talk between C5aR and TGFβ canonical and non-canonical pathway. C5aR, a G protein-coupled receptor for C5a anaphylotoxin, promotes the MAPK signaling activation (Ras/Raf/MEK), inducing the extracellular signal-regulated kinases (ERK) phosphorylation and transcription of pro-inflammatory and pro-fibrotic genes. pERK activation is also one of the final effector factors of SMAD-independent TGFβ pathway (non-canonical pathway) that include various branches of MAP kinase pathways, Rho-like GTPase signaling pathways and phosphatidylinositol-3-kinase/AKT pathways (*not showed*). Independently from TGFβ presence, C5a could activate SMAD-independent signaling leading to activation of profibrotic pathway. In addition, pERK could be involved in the activation of SMAD-dependent TGFβ pathway (canonical pathway, green arrow and factors) inducing the SMAD2/3 phosphorylation (red dotted arrow). As common downstream mechanisms, the C5a exposition led to transcription of profibrotic gene and proteinase for detachment. Blocking of pERK, by SC1 (Pluripotin) a dual kinase (ERK1, MAPK3) inhibitor of Thr-202/Tyr-204 phosphorylation could interferes with C5a-induced transcription of pro-inflammatory and with SMAD3 phosphorylation. This could lead to the decrease of extracellular matrix protein accumulation by perivascular pericytes (C5aR, complement component C5a Receptor 1; MAPK, mitogen-activated protein kinase, ERK, extracellular signal-regulated kinases; SMAD, small mother against decapentaplegic).

Figure S4 C5b-9 deposition occurred at peritubular capillary level and is modulated by C1-INH after 30 min of ischemia/reperfusion (I/R) injury. Immunofluorescence images showing interstitial peritubular capillaries pericytes co-labeled with PDGFRβ (green) and C5b-9 (red). In T0, PDGFRβ^+^/C5b-9^+^ perivascular cells were barely detectable **(A)**. After 30 min of I/R injury, the number of PDGFRβ^+^/C5b9^+^ cells increased predominately at peritubular capillaries level and glomerular level **(B)**. C1-INH treatment limited the C5b-9 deposition **(C)**. Results are expressed as media ± SEM of the numbers of PDGFRβ^+^/C5b-9^+^ cells/high power fields of five independent pigs for each group **(G)**. Scale bar in **(A–C)**: 100 µm. Boxed area was enlarged in **(D–F)** (scale bar: 50 µm).

## Abbreviations

I/R, ischemia/reperfusion; PMT, pericyte-to-myofibroblast transdifferentiation; C1-INH, C1-inhibitor; CTRL, control group; TGF-β1, transforming growth factor beta 1; PDGFRβ, beta-type platelet-derived growth factor receptor; NG2, neuronal glial antigen 2; Id2, inhibitor of DNA binding 2; ERK, extracellular signal-regulated kinases; SMAD, small mother against decapentaplegic; MMP9, matrix metallopeptidase 9; ADAMTS1, a disintegrin and metalloproteinase with thrombospondin motifs 1; CTGF, connective tissue growth factor; MAPK, mitogen-activated protein kinase.
