# Supplementary figures and images for: Complement Activation During Ischemia/Reperfusion Injury Induces Pericyte-to-Myofibroblast Transdifferentiation Regulating Peritubular Capillary Lumen Reduction Through pERK Signaling
Source: Front Immunol. 2018 May 23;9:1002. doi: 10.3389/fimmu.2018.01002 (PMC5974049; doi:10.3389/fimmu.2018.01002)

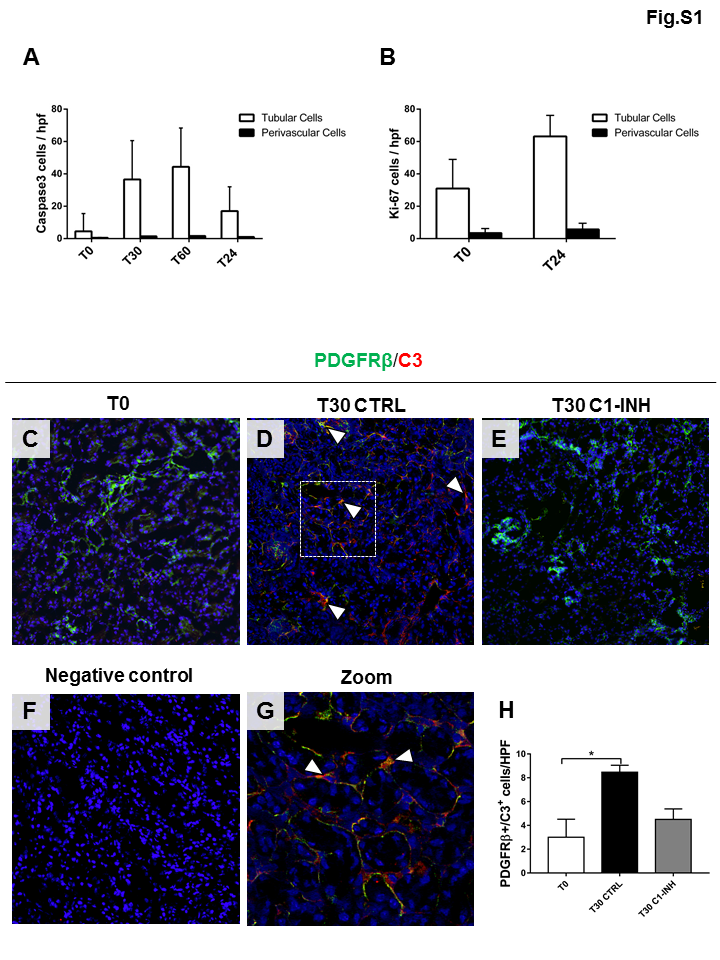

Supplement: Figure S1 — Ischemia/reperfusion (I/R) injury did not induce apoptosis or proliferation within perivascular compartment. Quantification of Casp3+ (A) and Ki-67+ (B) cells by IHC after I/R injury with distinction between tubular and perivascular cells. At different times from reperfusion, the Casp3+ and Ki-67+ were detected predominately at tubular level. Results are expressed as mean ± SD of Casp3+ or Ki-67+ cells/high power fields (HPF). Immunofluorescence images showing interstitial peritubular capillaries pericytes co-labeled with PDGFRβ (green) and C3 (red). In T0, PDGFRβ+/C3+ perivascular cells were barely detectable (C). After 30 min of I/R injury [(D), rectangle area was zoomed in (G)], the number of PDGFRβ+/C3+ cells increased predominately at peritubular capillaries level [(D), arrowheads]. C1-INH treatment limited the C3 deposition (E). Isotype control staining was used as negative control (F). Results are expressed as median ± interquartile range of the numbers of PDGFRβ+/C3+ cells/HPF of five independent pigs for each group (H). Magnification, 50×. [file image_1.tif]

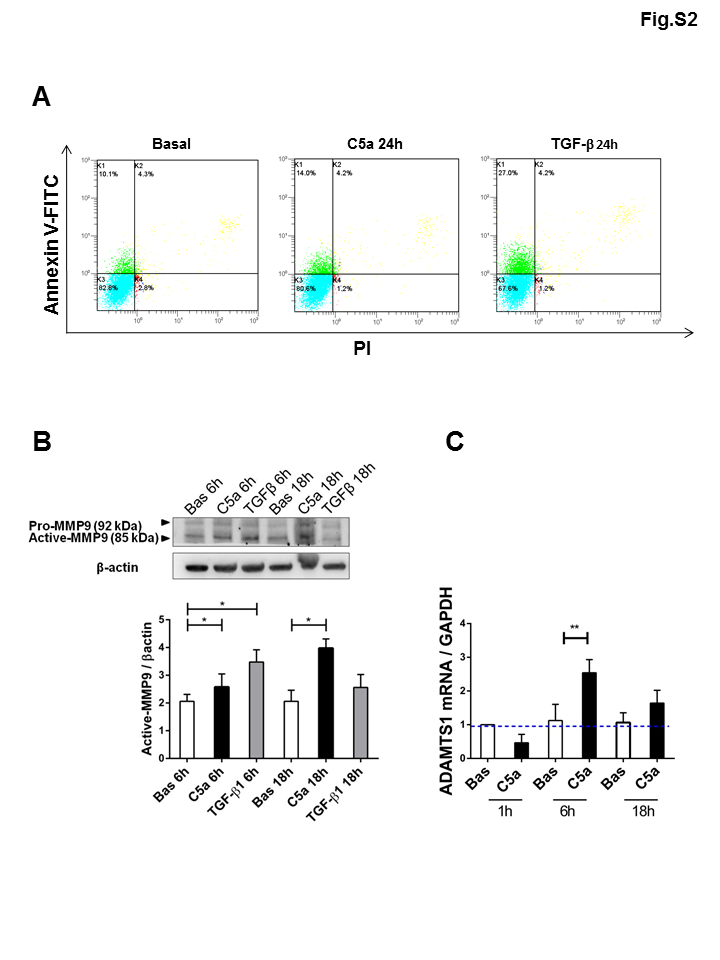

Supplement: Figure S2 — C5a did not trigger apoptosis and activated expression of matrix metallopeptidase (MMP9) and ADAMTS1 in pericytes. Human pericytes were treated with C5a and TGFβ for 6, 18, and 24 h. (A) Cell apoptosis or necrosis was analyzed by flow cytometry after Annexin V/propidium iodide (PI) staining. The units of the Y and X axes are fluorescence intensity. Early apoptotic cells are Annexin-V+/PI−; late apoptotic cells are both Annexin-V + 7/PI+; and necrotic cells were Annexin-V−/PI+. Data are expressed as apoptosis (early and late %) or necrosis (%) (as indicated in Figure 5C). (B) Western blotting demonstrated the increased expression of active form (85 kDa) of MMP9. (C) qPCR demonstrated the upregulation of ADAMTS1 mRNA after C5a and TGFβ exposition. p < 0.05 versus basal. [file image_2.tif]

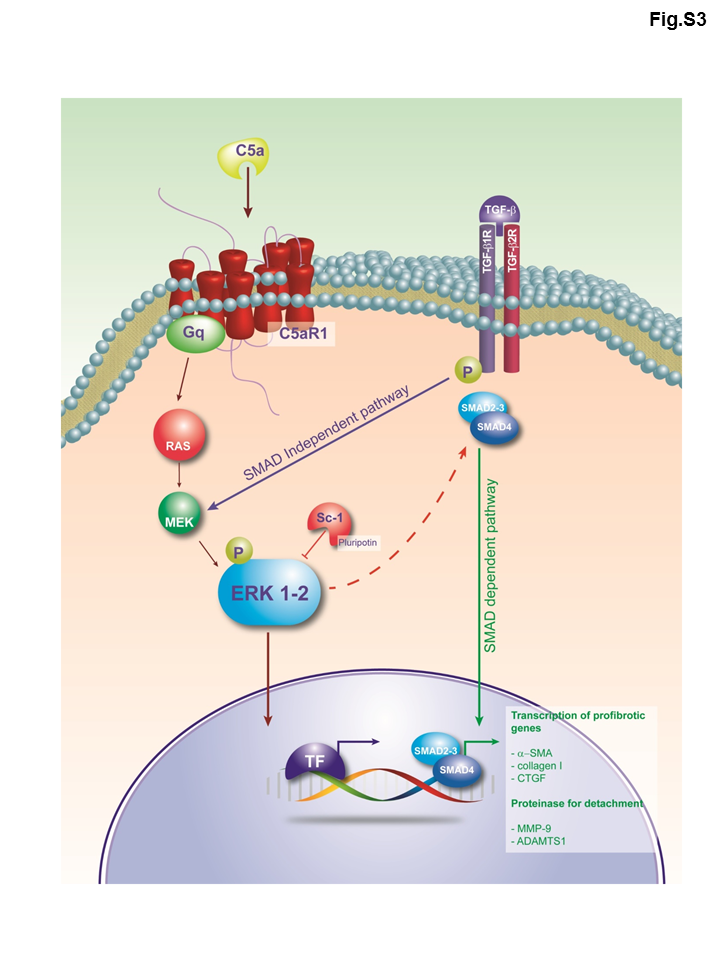

Supplement: Figure S3 — Schematic pathway showing the possible cross-talk between C5aR and TGFβ canonical and non-canonical pathway. C5aR, a G protein-coupled receptor for C5a anaphylotoxin, promotes the MAPK signaling activation (Ras/Raf/MEK), inducing the extracellular signal-regulated kinases (ERK) phosphorylation and transcription of pro-inflammatory and pro-fibrotic genes. pERK activation is also one of the final effector factors of SMAD-independent TGFβ pathway (non-canonical pathway) that include various branches of MAP kinase pathways, Rho-like GTPase signaling pathways and phosphatidylinositol-3-kinase/AKT pathways (not showed). Independently from TGFβ presence, C5a could activate SMAD-independent signaling leading to activation of profibrotic pathway. In addition, pERK could be involved in the activation of SMAD-dependent TGFβ pathway (canonical pathway, green arrow and factors) inducing the SMAD2/3 phosphorylation (red dotted arrow). As common downstream mechanisms, the C5a exposition led to transcription of profibrotic gene and proteinase for detachment. Blocking of pERK, by SC1 (Pluripotin) a dual kinase (ERK1, MAPK3) inhibitor of Thr-202/Tyr-204 phosphorylation could interferes with C5a-induced transcription of pro-inflammatory and with SMAD3 phosphorylation. This could lead to the decrease of extracellular matrix protein accumulation by perivascular pericytes (C5aR, complement component C5a Receptor 1; MAPK, mitogen-activated protein kinase, ERK, extracellular signal-regulated kinases; SMAD, small mother against decapentaplegic). [file image_3.tif]

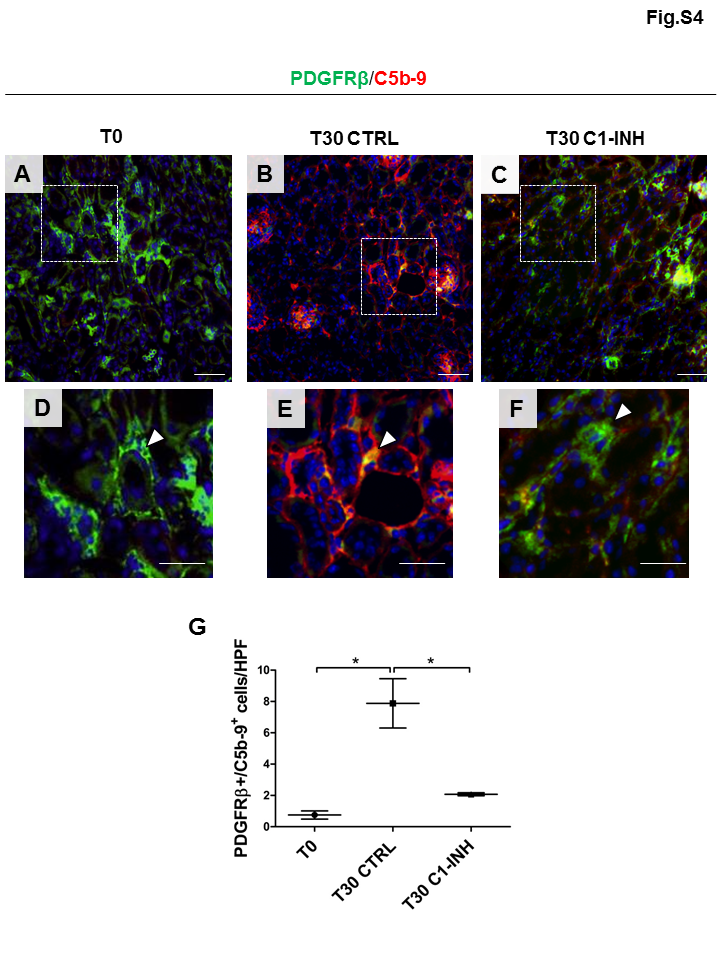

Supplement: Figure S4 — C5b-9 deposition occurred at peritubular capillary level and is modulated by C1-INH after 30 min of ischemia/reperfusion (I/R) injury. Immunofluorescence images showing interstitial peritubular capillaries pericytes co-labeled with PDGFRβ (green) and C5b-9 (red). In T0, PDGFRβ+/C5b-9+ perivascular cells were barely detectable (A). After 30 min of I/R injury, the number of PDGFRβ+/C5b9+ cells increased predominately at peritubular capillaries level and glomerular level (B). C1-INH treatment limited the C5b-9 deposition (C). Results are expressed as media ± SEM of the numbers of PDGFRβ+/C5b-9+ cells/high power fields of five independent pigs for each group (G). Scale bar in (A–C): 100 µm. Boxed area was enlarged in (D–F) (scale bar: 50 µm). [file image_4.tif]
